# Dynamics of Molecular Evolution and Phylogeography of *Barley yellow dwarf virus*-PAV

**DOI:** 10.1371/journal.pone.0016896

**Published:** 2011-02-04

**Authors:** Beilei Wu, Alexandra Blanchard-Letort, Yan Liu, Guanghe Zhou, Xifeng Wang, Santiago F. Elena

**Affiliations:** 1 State Key Laboratory for Biology of Plant Diseases and Insect Pests, Institute of Plant Protection, Chinese Academy of Agricultural Sciences, Beijing, People's Republic of China; 2 Instituto de Biología Molecular y Celular de Plantas, Consejo Superior de Investigaciones Científicas-Universidad Politécnica de Valencia, València, Spain; 3 The Santa Fe Institute, Santa Fe, New Mexico, United States of America; University of Wyoming, United States of America

## Abstract

*Barley yellow dwarf virus* (BYDV) species PAV occurs frequently in irrigated wheat fields worldwide and can be efficiently transmitted by aphids. Isolates of BYDV-PAV from different countries show great divergence both in genomic sequences and pathogenicity. Despite its economical importance, the genetic structure of natural BYDV-PAV populations, as well as of the mechanisms maintaining its high diversity, remain poorly explored. In this study, we investigate the dynamics of BYDV-PAV genome evolution utilizing time-structured data sets of complete genomic sequences from 58 isolates from different hosts obtained worldwide. First, we observed that BYDV-PAV exhibits a high frequency of homologous recombination. Second, our analysis revealed that BYDV-PAV genome evolves under purifying selection and at a substitution rate similar to other RNA viruses (3.158×10^−4^ nucleotide substitutions/site/year). Phylogeography analyses show that the diversification of BYDV-PAV can be explained by local geographic adaptation as well as by host-driven adaptation. These results increase our understanding of the diversity, molecular evolutionary characteristics and epidemiological properties of an economically important plant RNA virus.

## Introduction

RNA viruses are the most abundant parasites infecting humans, farm animals, and cultivated plants and are known for their high evolutionary potential [1]. This potential results from large mutation and recombination rates and, in the case of multipartite genomes, the re-assortment of segments during coinfection events. The high mutation frequencies generally observed in RNA virus populations are due to a fast rate of progeny production in the absence of proofreading mechanisms [2]. Recombination results from RNA-dependant RNA polymerase (RdRp) template-switching and represents a plastic genetic mechanism that may both contribute to speed up adaptation by bringing together beneficial mutations into the same genome or to facilitate the elimination of deleterious mutations by creating very low fitness genotypes carrying combinations of such mutations [3]. Furthermore, RNA viruses constitute an excellent experimental model to tackle general questions in evolutionary biology, such as for instance patterns of adaptive evolution, the effect of deleterious mutations accumulation in small populations, the evolution of specialists and generalist genotypes, the evolution of cooperation, or the evolution of genetic robustness, among many others [4].

Oswald & Houston [5] identified *Barley yellow dwarf virus* (BYDV) as a new positive sense ssRNA virus back in the mid of last century and it was subsequently found to have a worldwide distribution affecting nearly all members of the *Gramineae* [6]. Global yield losses due to the BYDVs are difficult to estimate because of insufficient information. However, average yield losses attributable to natural BYDV infection can range between 11 and 33% [6]; in some areas losses had been reported to reach up to 86%. Early work by Rochow and others distinguished five different strains of the virus by their primary aphid vector [7], [8]. Now, barley yellow dwarf viruses comprise BYDV-PAV, -MAV and -PAS species within the genus *Luteovirus*; *Cereal yellow dwarf virus*-RPV (CYDV-RPV, formerly BYDV-RPV) and CYDV-RPS in the genus *Polerovirus* as well as BYDV-SGV, -GPV and -RMV that have not yet been assigned to any genus [9]. Chinese isolates of BYDVs were divided into four species following Rochow's system, namely BYDV-GAV, -GPV, -PAV, and -RMV [10]. Virus isolates identified as BYDV-PAV were recently separated into three distinct subspecies, BYDV-PAV (PAV-I), -PAS (PAV-II) and PAV-CN (PAV-III), based in part on antibody reaction, genomic sequences, and/or symptoms in various host plants [11]. The separation into these three species was based upon the criteria that >10% differences at the amino acid level for any viral gene product discriminate between species within the *Luteoviridae* [11], [12].

The full genomes of 30 BYDV-PAV isolates collected from different regions of China during the period 2004 - 2006, were sequenced for this study (Table S1). In addition, we added 30 additional BYDV-PAV genomes from worldwide isolates available in GenBank (Table S2). Our aim is to generate a better description of the molecular evolutionary dynamics and epidemiology of BYDV-PAV. We first evaluated the impact of recombination in BYDV-PAV diversification. Then, we applied Bayesian Markov chain Monte Carlo (MCMC) coalescent analyses to estimate both the rate of nucleotide substitution and the time to the most common recent ancestor (TMRCA). Finally, also using MCMC methods, we analyzed whether the observed diversity could be explained by adaptation to local conditions or to host species.

## Results and Discussion

### Assessing the mosaic nature of BYDV-PAV isolates

**Figure 1:**
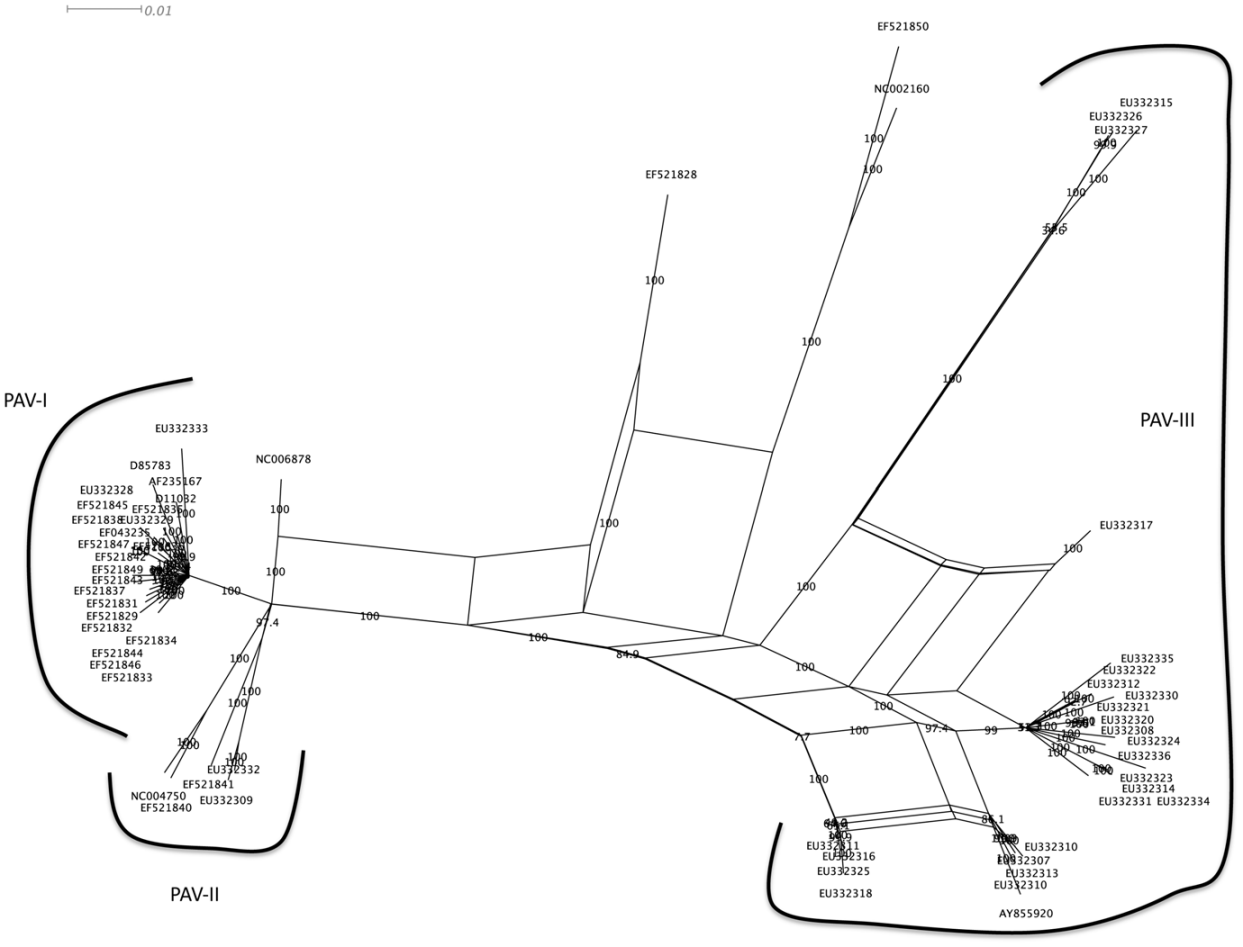
Split-decomposition phylogenetic network. Based on the 58 BYDV-PAV genomic sequences included in this study. Numbers over the branches represent bootstrap supports. The three PAV subspecies are indicated.

A split-decomposition network analysis (Figure 1) revealed evidence of conflicting phylogenetic signals within the 58 genomic sequences included in our study (Tables S1 and ). Many isolates can be connected to others by means of multiple evolutionary paths. This observation is not exclusive of geographically closer Chinese (PAV-III) isolates but also describes the evolution of worldwide PAV-I and PAV-II isolates. This intricate network-like pattern, rather than a purely bifurcating one, suggests extensive recombination during the diversification of BYDV-PAV species. A highly significant, *P*<0.001, pairwise homoplasy test [13] confirmed in a more quantitative manner the conclusion of reticulated evolution. Interestingly, isolate NC004750, classified as PAV-AUS significantly groups within the PAV-II subspecies.

Recombination is a pervasive phenomenon among BYDV-PAV isolates, as recently reported by others [14], [15] and confirmed here by all the methods used. A total of 22 unique recombination events were detected by at least five of the eight statistical methods implemented in RDP3. The statistical power of these methods strongly depends on the overall sequence divergence, being 5% the minimum necessary to attain significant power [16]. In our case, the overall pairwise sequence divergence was 14.4%, thus ensuring that the methods had enough statistical power to detect recombination breakpoints. Since several recombinant strains resulted from more than one recombination event, the final list of mosaic genomes had 18 entries. Table 1 shows the list of recombinant genomes, including the putative parental strains and the region involved in the exchange. Recombination breaking points appear distributed along the entire genome. Although we found many recombination breaking points in the boundaries of ORFs, we also found many putative points within coding sequences. Per ORF, the number of breaking points distributes as follows: 3 in UTR1, 17 in ORF1+2 (encoding for the RdRp), 3 in UTR2, 15 in ORF3(4)+5 (encoding for the RTP), 2 in ORF6 (encoding for the 7K protein), and 4 in UTR4. Pagán and Holmes [15] reported similar results when compared the three BYDV species (MAV, PAS and PAV). However, comparing the results of both studies, they found an excess of breaking points in gene boundaries compared with coding sequences, which is not our case. To conciliate this apparent discrepancy, we should keep in mind that by comparing different BYDV species, their sample should be bias towards viable interespecific recombinants that likely have exchanged full functional proteins. In our case, by looking into a single species, and given the higher sequence homology, viable recombinants may exist that have exchanged only parts of proteins.

**Table 1:** Recombinant BYDV-PAV strains.

Recombinant strain	Major parental	Minor parental	Region
EU332311	EU332310	EU332309	1–1365
EF521828	EF521850AJ810418	AJ810418EF521838	2733–55294956–5111
EU332317	EU332336EU332322	EU332327EU332315	1391–24013279–4137
AY855920	EU332307	EU332324	81–2931
EU332325	EU332335	EU332324	81–1350
EU332312	EU332322	EU332334	2834–4055
EU332314	EU332323	EU332334	2965–4162
EU332324	EU332335	EU332325	1371–2482
EU332335	EU332330	EU332312	2483–4261
AJ810418	NC004750NC004750NC004750	EF521836EF521850D11032	485–12542862–34914612–4979
NC004750	EU332333	D85783	1267–2853
EF521844	EU332332	EF521828	2110–2183
NC002160	EU332326	EF521835	2937–4451
EU332326	AF235167	EU332324	2482–3460
EU332315	AJ810418	EU332324	5132–5638
EU332322	EU332330	EU332317	163–1099
EU332310	AY855920	EU332313	2429–2936
EU332334	EU332330	EU332336	2853–4680

Most recombinant fragments encompass more than one ORF, for instance EU332335 resulted from the exchange of a region encompassing ORF2 and ORF3(4)+5 between isolates EU332330 and EU332312. In general, recombinant strains result from the exchange of a single fragment (i.e., a single recombination event). However, there are three isolates that result from two (EF521828 and EU332317) or even three (AJ810418) recombination events that generated highly mosaic genomes.

Among the observed 22 recombination events, the number of cases in which both parental strains were isolated from a common host, 13, was not significantly larger than the cases in which isolates were from different hosts, 9 (Binomial test, *P* = 0.524), suggesting that the host of isolation should not necessarily be the same in which the recombination event took place. Consistently, the resulting recombinant genome was found in the same host species than at least one of its parental in 21/22 cases, representing strain AJ810418 a particular case, since its region 2862–3491 came from viruses isolated from wheat and oat.

Regarding the geographic origin of the strains involved in recombination events, it is worth mentioning that in 12 cases the parental and recombinant strains shared a geographic origin, but that the remaining 10 cases did not share such origin, being the difference in counts not statistically significant (Binomial test, *P* = 0.832). This suggests that despite a significant geographic structure (see below), some genetic flow may still exist at the global scale. An interesting example of such diversity in geographic origins is the AJ810418 isolate, in whose origin were involved parental strains from Australia and from different locations within the USA. Consistent with this finding, BYDV movement between the USA and Australia as a consequence of maritime trade has been previously proposed after analyzing old gramineae samples conserved at herbaria [17]. In the following sections, the 18 recombinant strains have been removed from the analyses.

RNA viruses are renowned for their ability to evolve rapidly, a consequence of high mutation rates, rapid replication and large population sizes [2]. Moreover, there is mounting evidence for a role for recombination in shaping genetic diversity in some plant RNA viruses [18], [19]. Consequently, a greater understanding of the role of recombination in plant RNA virus evolution is of utmost importance. Recombination clearly plays a significant role in the evolution of RNA viruses by generating genetic variation, by reducing mutational load, and by producing new viruses [3]. Here, we have shown that BYDV-PAV exhibits a relatively high frequency of homologous recombination, explaining in part its evolutionary success.

### Evolutionary rate and age of genetic diversity

Our Bayesian coalescent estimates of evolutionary dynamics indicate that BYDV-PAV genome evolves at a rate of 3.158×10^−4^ substitutions per site and per year (95% HPD, 0.062−6.469×10^−4^). This substitution rate is within the range of values estimated for BYDV species and other luteoviruses [15] and, more generally, similar to values reported for other RNA viruses [20]. The Bayesian coalescent approach also allowed dating the origin of the PAV species of BYDV. We obtained an estimate of the TMRCA of 1741 years ago, although the estimate has a wide 95% HPD (from 268 to 4680 years ago). In their recent study, Pagán and Holmes [15] dated the TMRCA of the three BYDV species to be in the range 13–2009 years ago, depending on the particular gene analyzed (RdRp given more recent dates and RTD rendering older ones). Therefore, both estimates are in excellent agreement. Furthermore, the wide range 95% HPD we have obtained may simply reflect the heterogeneity in divergence times for the different ORFs in the BYDV-PAV genome.

### Selection analyses

Next, we sought to determine the selective forces that have been operating during the evolutionary diversification of BYDV-PAV. To do so, we used two standard approaches. The first approach was Tajima's *D* method to test whether the number of segregating sites in the sample significantly departs from the neutral expectation [21]. The first two columns of Table 2 show the *D* values and their associated statistical significance. The test suggests that RdRp (ORF1+2) and RTD (ORF3(4)+5) are evolving neutrally, whereas protein 7K (ORF6) may be under weak positive selection. However, departures from the null hypothesis can result from several different explanations (e.g., population subdivision or population expansion). Therefore, it is convenient to use an alternative test of neutrality.

**Table 2:** Results of the selection tests.

	Tajima's *D*	*P*	Negatively selected sites	Neutral sites	Positively selected sites
ORF1+2 (RdRp protein)	1.131	0.129	429	437	0
ORF3(4)+5 (RTD protein)	1.149	0.125	260	378	0
ORF6 (7K protein)	1.666	0.048	5	21	0

To overcome this drawback of Tajima's *D*, the second approach was to evaluate the difference in nonsynonymous and synonymous substitution rates per site, *d_N_* − *d_S_*. Table 2 shows the distribution of sites under purifying, neutral and directional selection for the three ORFs. Given that the same hypothesis (i.e., significant departure from the null neutral expectation) is tested multiple times on each ORF, we applied Bonferroni's sequential method to maintain an overall 5% significance level [22]. An Excel file containing the raw data can be requested from the authors. The picture drawn in Table 2 is one in which negative, or purifying, selection is the main evolutionary force. Not a single codon has been significantly evaluated as to be under positive selection. Codons under purifying selection are scattered along the ORFs, thus suggesting that the full protein, and not particular domains, are being constrained. The number of codons on each selective category differs among ORFs (homogeneity *χ* ^2^ = 18.727, 2 d.f., *P*<0.001). While RTD and 7K genes have more neutral codons than expected from the marginal distributions, the RdRp gene shows the opposite pattern: more codons are under purifying selection than expected. This finding confirms that the RdRp is the most functionally important protein of RNA viruses and hence, less prone to fix changes.

Therefore, we may conclude from these analyses that we have no enough statistical power as to identify any positively selected codon and, therefore, conservatively, we must conclude that current genetic diversity observed among BYDV-PAV isolates is compatible with the combined effects of purifying selection plus neutral evolution.

### Phylogeography of BYDV-PAV isolates

**Figure 2:**
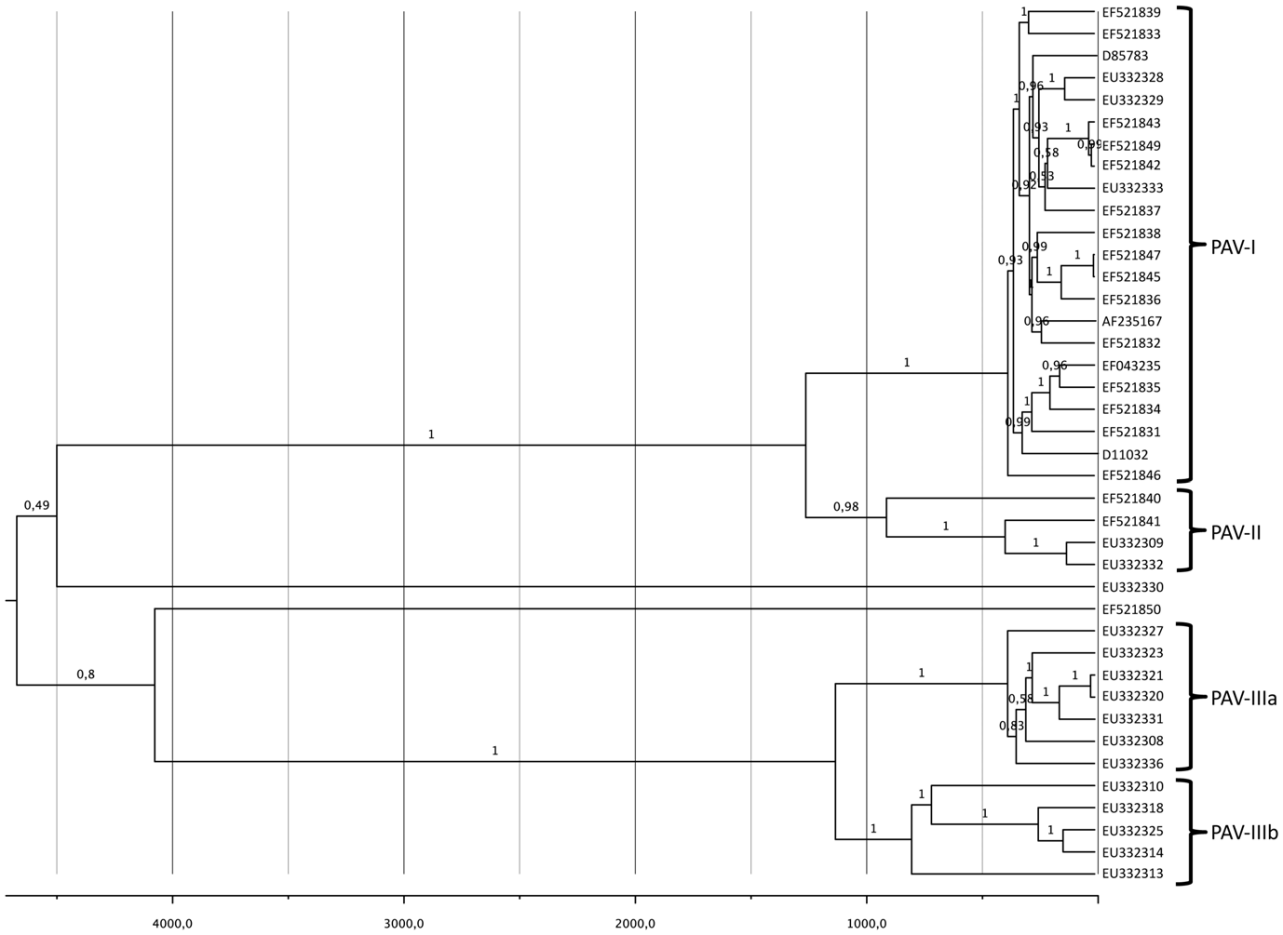
MCC phylogeny of 40 non-recombinant BYDV-PAV isolates. The tree was calculated from the posterior distribution of trees generated by Bayesian MCMC coalescent analysis with BEAST [32]. Posterior probabilities are indicated above branches.

Figure 2 shows the MCC tree obtained from the full genomes of the 40 isolates retained for this part of the study. The tree contains two large significant clades (posterior probabilities *P* = 1), and two isolates were placed outside these main clades, EU332330 and EF521850. Isolate EU332330 from China was previously assigned to PAV-III subspecies, whereas isolate EF521850 was assigned to PAV-I. At least two explanations can be brought forward to explain this misplacing. First, they were incorrectly classified and actually they belong to two new subspecies. Second, despite our effort to remove all recombinant genomes from our dataset, these two isolates have a mosaic origin and, consequently, they are placed at the basis of the clusters of their corresponding parentals. The first large clade contained all but one of the American isolates plus one European and five Chinese isolates. The clade can subsequently be divided into two significant sub-clades that correspond, respectively to the current definition of subspecies PAV-I (formerly PAV) and PAV-II (formerly PAS). Contrasting with this heterogeneity in geographic origin, the second large clade was only constituted by isolates of Chinese origin, all of them belonging to subspecies PAV-III (formerly PAV-CN). However, the PAV-III clade can subsequently be divided into two other significant subclades (posterior probabilities *P* = 1). In Figure 2 we named these two significant subclades as PAV-IIIa and PAV-IIIb. However, for the sake of consistency with the division in subspecies made in the first clade, the two PAV-III subclades could easily achieve the status of subspecies and be designated as PAV-III and PAV-IV.

Although the MCC tree is compatible with separating BYDV-PAV into 3–4 distinct subspecies based on full genomic information, the biological significance of these subspecies designations has yet to be determined. In general, all of the PAV-I and PAV-II isolates are recognized by anti-PAV polyclonal antibodies in serological assays [23] and by molecular diagnostic tools [24]. In addition, most isolates of BYDV-PAV were efficiently transmitted by *R. padi* and *S. avenae* but poorly so by *S. graminum* [25]. Symptoms of infection by BYDV-PAV consist typically of yellowing or reddening of leaves and stunted growth, though PAV-II isolates tend to induce more severe symptoms in host plants [24], however symptom severity varies across host species and even among cultivar of the same species [23], [24]. All of these evidences supported the genomic information of BYDV-PAV isolates may not accurately reflect the biology of a new species. Although different isolates of BYDV-PAV exhibit a divergence in genomic sequences, they share common biological features in symptom, vector transmission and so on. To avoid further confusion and complication of the literature, we would suggest that the rule to discriminate between species within the *Luteoviridae* based in a difference >10% at the amino acid level for any viral gene product should be modified and that more importance has to be given to differences in biological properties.

The phylogenetic MCC tree also suggests an apparent geographic structure in the genetic diversity of BYDV-PAV at the continental level, with the only exception of some PAV-I and PAV-II Chinese isolates that cluster according to subspecies (along with USA isolates) rather than according to geographic origin. To assess the strength of this structure in a more quantitative manner, we calculated three summary statistics (association index *AI*, parsimony score *PS* and maximum monophyletic clade size *MC*) describing the correlation between the geographic and phylogenetic relationships from the posterior distribution of genealogies generated by the Bayesian coalescence analysis. Table 3 shows the values and statistical significance for the three statistics. This analysis revealed the existence of significant signatures for geographic structure in the diversity of BYDV-PAV genomes when they were grouped by geographic origins (significant *AI* and *PS* values). This differentiation is mainly driven by the existence of two subpopulations, China and the USA (significant *MC* values). No inference was possible for the Japanese and European origins given their very limited sample size.

**Table 3:** Analysis of geographic and host effect on the population structure of BYDV-PAV isolates.

Analyses	Association statistics	Test value	*P*
Geographic regions	*PS*	5.7042	<0.001
	*AI*	0.2314	<0.001
China	*MC*	12.7983	0.001
Japan	*MC*	NA^1^	
Europe	*MC*	NA^1^	
America	*MC*	6.02310	0.017
Host species	*PS*	12.7998	0.001
	*AI*	1.6753	0.029
wheat	*MC*	12.7983	0.001
barley	*MC*	1.0149	1
oat	*MC*	2.0000	0.251
brome	*MC*	NA^1^	

1 Insufficient sample size (i.e., *n*<2).

Host-driven adaptation may also affect population structure of BYDV-PAV isolates. To check this possibility, we computed again the above statistics but using now host as the classification character. Table 3 also shows the values and significance of the three tests. A significant effect of host exists in the distribution of BYDV-PAV variability (significant *PS* and *AI* values). This effect is entirely driven by the wheat isolates (significant *MC* value), which cluster separately from the other three hosts (notice that no inference was possible for brome given only one isolate from this host was included in the study). However, some caution must be taken on this conclusion, as most of the isolates come from wheat, *AI* and *PS* might be significant just because most of the branches in the tree share the same state.

To summarize, we have shown that the evolutionary process in BYDV-PAV is shaped by a combination of very frequent recombination, relatively low rates of nucleotide substitution, itself a function of strong purifying selection operating on the three ORFs. BYDV-PAV shows evidences of population genetic structure both at the geographic level and, maybe, at the host level.

## Materials and Methods

### Virus propagation and purification

Along the growing seasons 2004–2006, 30 isolates of BYDV-PAV were collected during field surveys done in different agro-ecological areas from China, including the Northwestern (Shaanxi and Gansu provinces), Northern (Hebei province), Central (Hubei, Henan and Shandong provinces), and Southwestern (Yunan and Guizhou provinces) areas (Table S1). To increase virus concentration, every field isolate was inoculated to the susceptible host *Avena sativa L.* cv Coast Black by vectors *Rhopalosiphum padi*, *Sitobion avenae* and *Schizapus gramium*. Samples from each plant were screened for BYDV-PAV by DAS-ELISA using a specific antiserum (Bio-Rad). Leaves were collected from infection-positive plants displaying typical symptoms of BYDV-PAV infection, and stored at −80°C. Details of the isolates, their names, geographic origin, original host plant, and years of isolate are shown in Table S1.

### Cloning of entire genomes

Two weeks after inoculation, total RNAs were extracted from infected leaf samples with the Trizol Plant Mini Kit (Invitrogen), according to the manufacturer's protocol. RNA was reverse transcribed using the Promega cDNA synthesis system. Virus cDNA was amplified using AccuPrime™ Taq DNA Polymerase High Fidelity (Invitrogen), following the manufacturer's instructions and the primers of F1F/R, F2F/R, F3F/R, F4F/R and F5F/R (Table S3) that allowed the amplification of overlapping fragments encompassing the entire genome of BYDV-PAV. The PCR products were electrophoresed in 1.0% agarose gels, bands were excised using a razor blade and purified using the BioTeq PCR Quick Gel Extraction Kit (BioTeq Inc). The purified fragments were cloned into the pMD18-T vector (Takara). The plasmids were transformed into *Escherichia coli* strain JM110 and plasmid DNA was isolated from overnight cultures by alkaline analysis.

### DNA sequencing

Nucleotide sequences of the entire genome of each isolate were determined using the above PCR fragments. Insert sequences were determined on at least three clones for each PCR fragment using the dideoxynucleotide chain termination method by an ABI PRISM™ 3730 automated sequencer (Applied Biosystems). Overlapping sequences were assembled with program CONTIG from the STADEN version 2.0.0b7 package (staden.sourceforge.net). The nucleotide sequence data have been deposited in GenBank with accession numbers (EU332307-EU332336; Table S1).

### Preparing data for analyses

The BYDV- PAV genome is 5179–5709 nt in length and contains six open reading frames (ORFs) and four untranslated regions (UTRs) organized similarly to those of other members of the genus *Luteovirus* [26], [27]. ORF1 encodes for a protease. ORF2 encodes for the RNA-dependent RNA polymerase (RdRp) and it is expressed only fused to ORF1 via a low frequency −1 ribosomal frame shifting in the overlapping region. ORF3 encodes for the major coat protein (CP). ORF4, which is embedded within ORF3, is necessary for systemic movement of the virus throughout the phloem. Translation of ORF5 requires of an in-frame read-through of the ORF3 stop codon and the resulting peptide only exists fused to CP as a read-through domain (RTD). RTD is necessary for aphid transmission but not for virion assembly. ORF6 encodes for a small peptide (7K) of yet unknown function.

Complete genome sequences of the 30 BYDV-PAV isolates tested in this study plus other 28 BYDV-PAV (Table S2) isolates obtained from GeneBank were analyzed. In addition one sequence of BYDV-GAV and BYDV-MAV were included for the purpose of rooting the trees (NC004666 and NC003680, respectively; Table S2). For each ORF (1+2, 3(4)+5 and 6), the nucleotide sequences were translated and amino acids sequences aligned using MUSCLE [28]. The corresponding nucleotide alignments were obtained by concatenating codons using the REVTRANS server (http://www.cbs.dtu.dk/services/RevTrans; [29]). UTR sequences were individually aligned using MUSCLE. Full genome alignments were then obtained by concatenating all these partial alignments and used in all the analyses described below.

### Recombination analysis

Phylogenetic evidence for recombination was tested for the genomic sequences using the split-decomposition method implemented in SPLITSTREE version 4.11.3 [30] using the general time reversible (GTR) scheme of substitution with a gamma distribution (*Γ*_4_) model of site rates variation (GTR + *Γ*_4_) (see below for a justification of this choice). Branch support was evaluated by the bootstrap method based on 1000 pseudoreplicates. Putative recombination breakpoints were identified using the several methods implemented in RDP3 version 3.44b [31] with default configuration, except the options of linear sequence and of disentangling overlapping signals that were turned on. Only those genomes predicted to be recombinant by at least five methods where taken as valid. Recombinant genomes were removed from the dataset in all subsequent analyses.

### Estimating evolutionary dynamics

Prior to the Bayesian MCMC analyses, we first evaluated the existence of a temporal structure in our data set using PATH-O-GEN version 1.3 (tree.bio.ed.ac.uk/software/pathogen). This program used a Neighbor-joining phylogenetic tree (constructed using the GTR + *Γ*_4_ model of nucleotide substitutions) as input and performed a linear regression between the genetic distance from the root and the sampling time for each sequence. Genetic divergence significantly increased with time (*R* = 0.856), thus supporting the validity of the analyses described below.

Rates of molecular evolution and the TMRCA were estimated for the genomic alignments using the Bayesian MCMC approach implemented in the BEAST version 1.5.3 program [32]. The model of nucleotide substitution that best fitted the data was determined using MODELTEST version 3.7 [33]. Consequently, the data were analyzed using the GTR + *Γ*_4_ scheme of nucleotide substitution. In addition, different substitution rates were assigned to different codon positions. Substitution rates were estimated using a relaxed uncorrelated lognormal molecular clock [34]. This model had a Bayes factor 283 times larger than that defined by a strict molecular clock. A Uniform distribution in the range (0, 25 MYA) was used to calibrate the relaxed clock model. The upper value of the distribution corresponds to the estimated time of divergence between the *Avenaceae* and the *Triticaceae* [35]. We used the Bayesian skyline coalescent prior because it allows for both constant and complex changes in population size through time [36]. The MCMC was run for 10^8^ generations to ensure convergence of all parameters. TRACER version 1.5 (tree.bio.ed.ac.uk/software/tracer) was used to inspect posterior distributions and to estimate the relevant evolutionary parameters. The first 10% of sampled trees were discarded as burn-in. Statistical significance of parameters was evaluated using the 95% credible interval, also known as highest probability density (HPD).

### Phylogenetic analysis of geographic structure and host origin

The posterior set of trees produced from BEAST was used to estimate the maximum clade credibility (MCC) phylogeny, including its posterior probabilities. To do so, TREEANNOTATOR version 1.5.3 (beast.bio.ed.ac.uk) was employed with a 10% of the trees discarded as burn-in. 95% HPD confidence intervals were used to evaluate the reliability of the MCC tree. To determine the extent of geographic structure in BYDV-PAV populations, we used BATS version 1.0b2 [37] to compute the parsimony score (*PS* [38]), the association index (*AI* [39]) and the maximum monophyletic clade size (*MC* [37]) statistics and to assess their significance. The first 10% of sampled trees were discarded as burn-in and 10^4^ randomizations were performed to estimate the null distributions of the three statistics.

### Estimation of selection pressures

Selective pressures on each codon were evaluated using the difference between nonsynonymous (*d_N_*) and synonymous (*d_S_*) substitution rates per codon using the single-likelihood ancestor counting (SLAC), fixed-effects likelihood (FEL) and internal branches fixed-effects likelihood (IFEL) methods implemented in the HYPHY version 2.0 package [40]. Values of *d_N_* − *d_S_*<0,  = 0 or >0 indicate negative selection, neutral evolution and positive selection, respectively. Estimates of the difference in substitution rates were made from a phylogenetic tree inferred using the Neighbor-Joining algorithm with distances corrected under the GTR+*Γ*_4_ model.

## Supporting Information

Table S1 Isolates of BYDV-PAV characterized for this study.
(DOC)

Table S2 BYDV-PAV sequences from GenBank that have been included in this study.
(DOC)

Table S3 Synthetic oligonucleotide primers used for RT-PCR amplification and corresponding annealing temperatures.
(DOC)

## References

[1] Elena SF, Sanjuán R (2008). Virus evolution, insights from an experimental approach.. Annu Rev Ecol Evol Syst.

[2] Sanjuán R, Nebot MR, Chirico N, Mansky LM, Belshaw R (2010). Viral mutation rates.. J Virol.

[3] Worobey M, Holmes EC (1999). Evolutionary aspects of recombination in RNA viruses.. J Gen Virol.

[4] Elena SF, Agudelo-Romero P, Carrasco P, Codoñer FM, Martín S (2008). Experimental evolution of plant RNA viruses.. Heredity.

[5] Oswald JW, Houston BR (1951). A new virus disease for cereals, transmissible by aphids.. Plant Dis Rep.

[6] Miller MA, Rasochová L (1997). Barley yellow dwarf viruses.. Annu Rev Phytopathol.

[7] Rochow WF (1969). Biological properties of four *isolates of Barley yellow dwarf virus*.. Phytopathology.

[8] Rochow WF, Muller I (1971). Fifth variant of *Barley yellow dwarf virus* in New York.. Plant Dis Rep.

[9] Van Regenmortel MH, Mayo MA, Fauquet CM, Maniloff J (2000). Virus nomenclature: consensus versus chaos.. Arch Virol.

[10] Zhou GH, Zhang SX, Qian YT (1987). Identification and applications of four strains of *Wheat yellow dwarf virus*.. Sci Agric.

[11] Liu F, Wang X, Liu Y, Xie J, Gray SM (2007). A Chinese isolate of *Barley yellow dwarf virus*-PAV represents a third distinct species within the PAV serotype.. Arch Virol.

[12] D'Arcy CJ, Domier LL, Fauquet CM, Mayo MA, Maniloff J, Desselberger U, Ball LA (2005). Family *Luteoviridae*:. Virus Taxonomy. VIIIth Report of International Committee on Taxonomy of Viruses.

[13] Bruen TC, Philippe H, Bryant D (2006). A simple test for detecting the presence of recombination.. Genetics.

[14] Boulila M (2011). Selective constraints, molecular recombination structure and phylogenetic reconstruction of isometric plant RNA viruses of the families *Luteoviridae* and *Tymoviridae*.. Biochime.

[15] Pagán I, Holmes EC (2010). Long-term evolution of the *Luteoviridae*: time scale and mode of virus speciation.. J Virol.

[16] Posada D, Crandall KA (2001). Evaluation of methods for detecting recombination from DNA sequences: computer simulations.. Proc Natl Acad Sci USA.

[17] Malmstrom CM, Shu R, Linton EW, Newton LA, Cook MA (2007). Barley yellow dwarf viruses (BYDVs) preserved in herbarium specimens illuminate historical disease ecology of invasive and native grasses.. J Ecol.

[18] Chare ER, Holmes EC (2006). Phylogenetic survey of recombination frequency in plant RNA viruses.. Arch Virol.

[19] Codoñer FM, Elena SF (2008). The promiscuous evolutionary history of the family *Bromoviridae*.. J Gen Virol.

[20] Duffy S, Shackelton LA, Holmes EC (2008). Rates of evolutionary change in viruses: patterns and determinants.. Nat Rev Genet.

[21] Tajima F (1989). Statistical method for testing the neutral mutation hypothesis by DNA polymorphism.. Genetics.

[22] Rice WR (1989). Analyzing tables of statistical tests.. Evolution.

[23] Chay CA, Smith DM, Vaughan R, Gray SM (1996). Diversity among isolates within the PAV serotype of *Barley yellow dwarf virus*.. Phytopathology.

[24] Mastari J, Lapierre H, Dessens JT (1998). Asymmetrical distribution of *Barley yellow dwarf virus* PAV variants between host plant species.. Phytopathology.

[25] Du ZQ, Li L, Liu L, Wang XF, Zhou G (2007). Evaluation of aphid transmission abilities and vector transmission phenotypes of barley yellow dwarf viruses in China.. J Plant Pathol.

[26] Mayo MA, Ziegler-Graff V (1996). Molecular biology of luteoviruses.. Adv Virus Res.

[27] Miller WA, Liu S, Bekett R (2002). *Barley yellow dwarf virus*: *Luteoviridae* or *Tombusviridae*?. Mol Plant Pathol.

[28] Edgar R (2004). MUSCLE: Multiple sequence alignment with high accuracy and throughput.. Nucl Acids Res.

[29] Wernersson R, Pedersen AG (2003). RevTRans – constructing alignments of coding DNA from aligned amino acids sequences.. Nucl Acids Res.

[30] Huson DH, Bryant D (2006). Application of phylogenetic networks in evolutionary studies.. Mol Biol Evol.

[31] Martin DP, Lemey P, Lott M, Moulton V, Posada D (2010). RDP3: a flexible and fast computer program for analyzing recombination.. Bioinformatics.

[32] Drummond AJ, Rambaut A (2007). BEAST: Bayesian evolutionary analysis by sampling trees.. BMC Evol Biol.

[33] Posada D, Crandall KA (2001). Selecting the best-fit model of nucleotide substitution.. Syst Biol.

[34] Drummond AJ, Ho SYW, Phillips MJ, Rambaut A (2006). Relaxed phylogenetics and dating with confidence.. PLoS Biology.

[35] Gaunt BS (2002). Evolutionary dynamics of grass genomes.. New Phytol.

[36] Drummond AJ, Rambaut A, Shapiro B, Pybus OG (2005). Bayesian coalescent inference of past population dynamics from molecular sequences.. Mol Biol Evol.

[37] Parker J, Rambaut A, Pybus OG (2008). Correlating viral phenotypes with phylogeny: accounting for phylogenetic uncertainty.. Infect Genet Evol.

[38] Slatkin M, Maddison WP (1989). A cladistic measure of gene flow measured from phylogenies of alleles.. Genetics.

[39] Wang TH, Donaldson YK, Brettle RP, Bell JE, Simmonds P (2001). Identification of shared populations of *Human immunodeficiency virus* type 1 infecting microglia and tissue macrophages outside the central nervous system.. J Virol.

[40] Kosakovsky Pond SL, Frost SDW, Muse SV (2005). HYPHY: hypothesis testing using phylogenies.. Bioinformatics.

