# Supplementary material for: Dynamics of Molecular Evolution and Phylogeography of Barley yellow dwarf virus-PAV
Source: PLoS One. 2011 Feb 4;6(2):e16896. doi: 10.1371/journal.pone.0016896 (PMC3033904; doi:10.1371/journal.pone.0016896)
Supplement: Table S1 — Isolates of BYDV-PAV characterized for this study. (DOC) [file pone.0016896.s001.doc]

**Table S1** Isolates of BYDV-PAVs characterized for this study.

| **Accession numbers** | **Isolate** | **Geographical Origin** | | **Collection time** | **Host** |
| --- | --- | --- | --- | --- | --- |
|  |  | **Province** | **City** |  |  |
| EU332317 | BYDV/wheat/Shaanxi/05YL5/2005/PAV-III | Shannxi | Yangling | 2005 | Wheat |
| EU332318 | BYDV/wheat/Shaanxi/05YL8/2005/PAV-III |  |  |  |  |
| EU332310 | BYDV/wheat/Shaanxi/05YL10/2005/PAV-III |  |  |  |  |
| EU332315 | BYDV/wheat/Shaanxi/05WN1/2005/PAV-III | Shannxi | Weinan | 2005 | Wheat |
| EU332316 | BYDV/wheat/Shaanxi/05WN6/2005/PAV-III |  |  |  |  |
| EU332332 | BYDV/wheat/Yunnan/06KM14/2006/PAV-II | Yunnan | Kunming | 2006 | Wheat |
| EU332333 | BYDV/wheat/Yunnan/06KM25/2006/PAV-I |  |  |  |  |
| EU332328 | BYDV/wheat/Guizhou/06GY1/2006/PAV-I | Guizhou | Guiyang | 2006 | Wheat |
| EU332329 | BYDV/wheat/Guizhou/06GY5/2006/PAV-I |  |  |  |  |
| EU332313 | BYDV/wheat/Gansu/05TS3/2005/PAV-III | Gansu | Tianshui | 2005 | Wheat |
| EU332309 | BYDV/wheat/Gansu/05GG2/2005/PAV-II | Gansu | Gangu | 2005 | Wheat |
| EU332310 | BYDV/wheat/Gansu/05GG5/2005/PAV-III |  |  |  |  |
| EU332311 | BYDV/wheat/Gansu/05GG6/2005/PAV-III |  |  |  |  |
| EU332308 | BYDV/wheat/Henan/04ZZ5/2004/PAV-III | Henan | Zhengzhou | 2004 | Wheat |
| EU332320 | BYDV/wheat/Henan/05ZZ1/2005/PAV-III |  |  | 2005 | Wheat |
| EU332321 | BYDV/wheat/Henan/05ZZ4/2005/PAV-III |  |  |  |  |
| EU332322 | BYDV/wheat/Henan/05ZZ6/2005/PAV-III |  |  |  |  |
| EU332323 | BYDV/wheat/Henan/05ZZ7/2005/PAV-III |  |  |  |  |
| EU332324 | BYDV/wheat/Henan/05ZZ9/2005/PAV-III |  |  |  |  |
| EU332325 | BYDV/wheat/Henan/05ZZ10/2005/PAV-III |  |  |  |  |
| EU332326 | BYDV/wheat/Henan/05ZZ12/2005/PAV-III |  |  |  |  |
| EU332327 | BYDV/wheat/Henan/05ZZ13/2005/PAV-III |  |  |  |  |
| EU332335 | BYDV/wheat/Henan/06ZZ4/2006/PAV-III |  |  | 2006 | wheat |
| EU332336 | BYDV/wheat/Henan/06ZZ5/2006/PAV-III |  |  |  |  |
| EU332307 | BYDV/wheat/Henan/06ZZ10/2006/PAV-III |  |  |  |  |
| EU332312 | BYDV/wheat/Henan/05JZ4/2005/PAV-III | Henan | Jiaozuo | 2005 | Wheat |
| EU332314 | BYDV/wheat/Hubei/05WH6/2005/PAV-III | Hubei | Wuhan | 2005 | Weeds |
| EU332334 | BYDV/wheat/Hubei/06WH1/2006/PAV-III |  |  | 2006 | Wheat |
| EU332330 | BYDV/wheat/Shandong/06JN2/2006/PAV-III | Shandong | Jinan | 2006 | Wheat |
| EU332331 | BYDV/wheat/Shandong/06JN4/2006/PAV-III |  |  |  |  |
