# Supplementary material for: Dynamics of Molecular Evolution and Phylogeography of Barley yellow dwarf virus-PAV
Source: PLoS One. 2011 Feb 4;6(2):e16896. doi: 10.1371/journal.pone.0016896 (PMC3033904; doi:10.1371/journal.pone.0016896)
Supplement: Table S2 — BYDV-PAV sequences from GenBank that have been included in this study. (DOC) [file pone.0016896.s002.doc]

**Table S2** BYDV-PAV sequences from GenBank that have been included in this study.

| **Accession**  **Number** | **Isolate** | **Geographical Origin**  **(Country/Region)** | | **Host** | **Approximate**  **collection year** |
| --- | --- | --- | --- | --- | --- |
| AY855920 | BYDV/wheat/China/AY855920/2004/PAV-III | China |  | Wheat | 2004 |
| NC004750 | BYDV/Australia/NC004750/1988/PAV-AUS | Australia |  |  | 1988 |
| EF521850 | BYDV/oat/Alaska/064/2007/PAV-I | USA | Alaska | Oat | 2007 |
| EF521849 | BYDV/California/0102/2007/PAV-I | USA | California |  | 2007 |
| EF521847 | BYDV/Iowa/050/2007/PAV-I | USA | Iowa |  | 2007 |
| EF521846 | BYDV/oat/Alaska/068/2007/PAV-I | USA | Alaska | Oat | 2007 |
| EF521845 | BYDV/Arkansas/045/2007/PAV-I | USA | Arkansas |  | 2007 |
| EF521844 | BYDV/barley/Iowa/014/2007/PAV-I | USA | Iowa | Barley | 2007 |
| EF521843 | BYDV/California/0100/2007/PAV-I | USA | California |  | 2007 |
| EF521842 | BYDV/California/0101/2007/PAV-I | USA | California |  | 2007 |
| EF521841 | BYDV/Sweden/052/2007/PAV-I | Sweden |  |  | 2007 |
| EF521840 | BYDV/Oregon/048/2007/PAV-I | USA | Oregon |  | 2007 |
| EF521838 | BYDV/New York/047/2007/PAV-I | USA | New York |  | 2007 |
| EF521837 | BYDV/California/043/2007/PAV-I | USA | California |  | 2007 |
| EF521836 | BYDV/Oklahoma/020/2007/PAV-I | USA | Oklahoma |  | 2007 |
| EF521835 | BYDV//Iowa/016/2007/PAV-I | USA | Iowa | Oat | 2007 |
| EF521834 | BYDV/oat/Iowa/013/2007/PAV-I | USA | Iowa | Oat | 2007 |
| EF521833 | BYDV/oat/Iowa/015/2007/PAV-I | USA | Iowa | Oat | 2007 |
| EF521832 | BYDV/oat/Iowa/012/2007/PAV-I | USA | Iowa | Oat | 2007 |
| EF521831 | BYDV/wheat/Missouri/011/2007/PAV-I | USA | Missouri | Wheat | 2007 |
| EF521829 | BYDV/brome/Missouri/005/2007/PAV-I | USA | Missouri | Brome | 2007 |
| EF521828 | BYDV/barley/Iowa/0109/2007/PAV-I | USA | Iowa | Barley | 2007 |
| EF043235 | BYDV/wheat/Kansas/003/2006/PAV-I | USA | Kansas | Wheat | 2006 |
| NC002160 | BYDV/USA/NC002160/1999/PAV-II | USA |  |  | 1999 |
| AJ810418 | BYDV/barley/Germany/AJ810418/2004/PAV-ASL | Germany |  | Barley | 2004 |
| D85783 | BYDV/barley/Japan/D85783/1996/PAV-JPN | Japan |  | Barley | 1996 |
| D11032 | BYDV/USA/D11032/1990/PAV-P | USA |  |  | 1990 |
| AF235167 | BYDV/USA/AF235167/2000/PAV-I | USA |  |  | 2000 |
| NC004666 | BYDV/wheat/China/NC004666/2003/GAV | China |  | Wheat | 2003 |
| NC003680 | BYDV/USA/NC003680/1990/MAV | USA |  |  | 1990 |
