# Supplementary material for: Dynamics of Molecular Evolution and Phylogeography of Barley yellow dwarf virus-PAV
Source: PLoS One. 2011 Feb 4;6(2):e16896. doi: 10.1371/journal.pone.0016896 (PMC3033904; doi:10.1371/journal.pone.0016896)
Supplement: Table S3 — Synthetic oligonucleotide primers used for RT-PCR amplification and corresponding annealing temperatures. (DOC) [file pone.0016896.s003.doc]

**Table S3**  Synthetic oligonucleotide primers used for RT-PCR amplification and corresponding annealing temperatures.

| Primer | Nucleotide Sequence(5’→3’) | Position* | Annealing Temperature （°C） |
| --- | --- | --- | --- |
| F1F | 5‘- TGAAGATTGACCATCTCAC -3’ | 1-19nt | 54 |
| F1R | 5‘-TGTCTTGCAGTATCCCACCT -3’ | 1476-1457nt | 54 |
| F2F | 5‘- TTTACAGTAGGAAAGGGGGA -3’ | 1357-1376nt | 55 |
| F2R | 5‘- GAAGCTTTCGAGTATGGGTAC -3’ | 2433-2413nt | 55 |
| F3F | 5‘- ATGTCGGCTGTGGCTCAGTG -3’ | 2371-2390nt | 55 |
| F3R | 5‘- TTGAGGAGTCTACCTATTTGG -3’ | 3468-3448nt | 55 |
| F4F | 5‘- TAGCTACATTAACTCATTCAC -3’ | 3263-3283nt | 52 |
| F4R | 5‘- GCCTCAACATCGGATTCATC -3’ | 4177-4158nt | 52 |
| F5F | 5‘- CTATGTTGTCTCGTATGGAGG -3’ | 4091-4111nt | 55 |
| F5R | 5‘- GGGATGCCGAACTTCTCTTGC -3’ | 5652-5632nt | 55 |
